# CD40-TRAF6 inhibition suppresses cardiovascular inflammation, oxidative stress and functional complications in a mouse model of arterial hypertension

**DOI:** 10.1016/j.redox.2025.103520

**Published:** 2025-01-29

**Authors:** Lea Strohm, Henning Ubbens, Dominika Mihalikova, Alexander Czarnowski, Paul Stamm, Michael Molitor, Stefanie Finger, Matthias Oelze, Dorothee Atzler, Philip Wenzel, Philipp Lurz, Thomas Münzel, Christian Weber, Esther Lutgens, Andreas Daiber, Steffen Daub

**Affiliations:** a Department of Cardiology, Cardiology I, University Medical Center of the Johannes Gutenberg-University, Mainz, Germany; b Center for Thrombosis and Hemostasis, University Medical Center of the Johannes Gutenberg-University, Mainz, Germany; c German Center for Cardiovascular Research (DZHK), Partnersite Rhine-Main, Mainz, Germany; d Institute for Cardiovascular Prevention, Ludwig-Maximilians-Universität München, Munich, Germany; e DZHK (German Center for Cardiovascular Research), Partner Site Munich Heart Alliance, Germany; f Walter Straub Institute of Pharmacology and Toxicology, Ludwig-Maximilians-Universität, Munich, Germany; g Munich Cluster for Systems Neurology (SyNergy), Munich, Germany; h Mayo Clinic, Dept Cardiovascular Medicine and Immunology, Rochester, MN, USA

**Keywords:** Arterial hypertension, Endothelial function, Blood pressure, Inflammation, CD40(L)-TRAF6 signaling

## Abstract

Cardiovascular disease is the leading cause of disease burden and death worldwide and is fueled by vascular inflammation. CD40L–CD40–TRAF signaling is involved in the progression of atherosclerosis and drives the development of coronary heart disease (CHD). The present study investigates whether the CD40L-CD40-TRAF6 signaling pathway with focus on immune cells and adipocytes could be a therapeutic target in arterial hypertension.

Arterial hypertension was induced in WT (C57BL6/J) and cell-specific CD40(L) knockout mice (AdipoqCre x CD40 fl/fl, CD4Cre x CD40 fl/fl, CD19Cre x CD40 fl/fl, and GP1baCre x CD40L fl/fl) via angiotensin (AT-II) infusion (1 mg/kg/d) for seven days. Hypertensive WT mice were also treated with a CD40-TRAF6 inhibitor (2.5 mg/kg/d, for 7d). The TRAF6 inhibitor treatment normalized endothelial dysfunction and reduced blood pressure in hypertensive wild type animals. Reactive oxygen species production was decreased by TRAF6 inhibition in blood, aorta, heart, kidney, and perivascular fat tissue. Additionally, FACS analysis revealed that TRAF6 inhibition prevents immune cell migration into the aortic vessel wall observed by reduced CD45^+^ leukocyte, Ly6G^+^/Ly6C^+^ neutrophil, and Ly6C^high^ inflammatory monocyte content. The hypertensive cell type-specific CD40(L) knockout animals showed only a minor effect on endothelial function, blood pressure, and oxidative stress. Therefore, we conclude that targeting CD40 directly on adipocytes, B-cells, T-cells, or CD40L on platelets is not a promising target to prevent hypertension complications.

In summary, TRAF6 inhibition but not adipocyte, B-cell, or T-cell-specific CD40 or platelet-specific CD40L deficiency reduces pathophysiological vascular inflammation in hypertensive mice, suggesting TRAF6 inhibition as a potential therapeutic target in hypertensive patients.

## Highlights

• Hypertension if the leading risk factor for global deaths.
• CD40-TRAF6 inhibition represent a novel target for antihypertensive therapy.
• TRAF6 inhibition normalized blood pressure and endothelial dysfunction.
• TRAF6 inhibition prevented oxidative stress and inflammation in different tissues by reduced vascular infiltration of leukocytes.
• TRAF6 inhibition slightly improved cardiac function (ejection fraction).

## Graphical abstract

**Figure:**
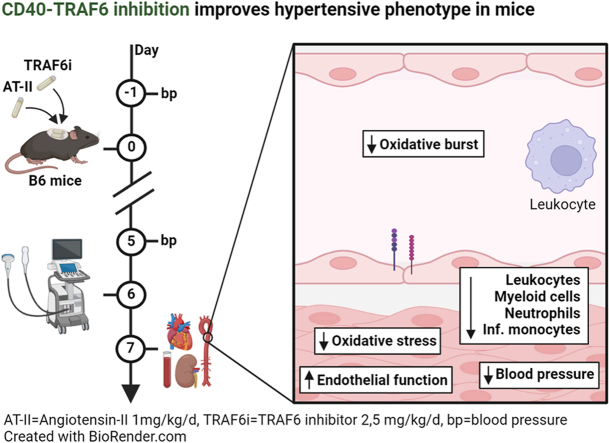

## 1 Introduction

The CD40L–CD40 co-stimulatory interaction plays a significant role in the development of cardiovascular disease [1] and immunomodulation represents an attractive therapeutic target in cardiovascular disease [2]. CD40L and CD40 belong to the TNF-receptor superfamily. CD40 has no intrinsic signaling capabilities and needs small adaptor proteins termed “TNF receptor-associated factors” (TRAFs) for its signal transduction. TRAF2/3/5 are binding distal to the intracellular domain of CD40, whereas TRAF6 is binding more proximal [3].

Blocking CD40L decreases atherosclerosis and promotes plaque stability in hyperlipidemic mice by dampening key inflammatory pathways [4]. Similarly, CD40L knockout helps to prevent oxidative stress, inflammation, and endothelial dysfunction in mice with hypertension, diabetes, or obesity [4,5]. In contrast to the CD40L knockout model, global CD40 knockout aggravates inflammation and insulin resistance in obese mice [6]. At the same time, endothelial cell-specific CD40 deficiency proved protective in atherosclerotic apolipoproteinE (ApoE)^−/−^ mice [7].

A comprehensive blockade of CD40L or CD40 signaling does not appear to be a viable long-term treatment for atherosclerosis, mainly because of the undesirable side effects of immunosuppression or the risk of thromboembolic events [3]. On the other hand, targeting downstream molecules in the CD40 signaling pathway has shown to be both effective and safe for treating cardiovascular disease [6,8,9]. A small molecule inhibitor focusing on CD40-TRAF6 signaling can prevent atherosclerosis without leading to immune suppression [10]. Recently, our group has investigated the role of CD40-CD40L-TRAF6 signaling in patients suffering from coronary heart disease and accompanied by arterial hypertension and/or diabetes and found comparable pathways in respective mouse models [11]. Our present study aimed to detail the effects of CD40L-CD40-TRAF6 signaling in hypertensive mice using a CD40-TRAF6 small molecule inhibitor in a model of arterial hypertension with a key role for reactive oxygen species formation by phagocytic NADPH oxidase [[12], [13], [14]]. Further, the role of different cell types in CD40(L) driven pro-inflammatory signaling was investigated. We used different genetic mouse models of specific CD40 deficiency on adipocytes, B-, and T-cells to assess the impact on hypertension. Finally, a platelet-specific CD40L knockout was investigated using the same model of hypertension.

## 2 Methods

### 2.1 Animals and treatment

All animal procedures within this study followed the Guide for the Care and Use of Laboratory Animals as adopted and promulgated by the US National Institute of Health. Our animal studies were approved by the Ethics Committee of the University Hospital Mainz and the Landesuntersuchungsamt Rheinland-Pfalz (Koblenz, Germany, G19-1-068 and extensions 1–3). The animals were housed in a standard 12-h light-dark cycle with ad libitum access to standard rodent chow and water according to the Translational Animal Research Center (TARC) guidelines of the University Medical Center in Mainz. Animal experiments were performed at the same time of the day to minimize circadian variation.

Male C57BL/6J (9–10 weeks old) mice were purchased from Charles River (Sulzfeld, Germany) and treated with either angiotensin-II (AT-II; 1 mg/kg/d in 0.9 % NaCl) [11,15], TRAF6 inhibitor (compound 6877002, Tocris, Bristol, UK; 2.5 mg/kg/d in 2.5 % DMSO) [4,11] or both compounds by osmotic minipumps (Alzet model 1007D, Cupertino, CA, USA) for seven days. The administration of AT-II and TRAF6 inhibitors were performed in separate pumps because of the different solubility of the drugs. The pumps were implanted s.c. under ketamine/xylazine anesthesia (120 mg/kg; 16 mg/kg; injected fluid volume 0.1–0.2 ml). The mice were killed under anesthesia by opening the thorax and rapid exsanguination by heart puncture. The treatment was performed as previously described [4,11]. In total, 16 mice were used per group.

Adipocyte CD40-deficient mice (AdipoqCre x CD40 fl/fl or AdiCD40^−/−^) were initially generated by crossbreeding CD40 fl/fl mice [16] with AdipoQCre mice (Jackson Laboratories, Bar Harbour, Main, USA) strain B6.FVB-Tg-1Evdr/J) as described in Ref. [17]. B-cell CD40-deficient mice (CD19Cre x CD40 fl/fl or CD19Cre) were generated by crossbreeding CD40 fl/fl mice [16] with Cd19tm1(cre)Cgn mice (Jackson Laboratories - Stock No: 018958). T-cell CD40-deficient mice (CD4Cre x CD40 fl/fl or CD4Cre) were generated by crossbreeding CD40 fl/fl mice [16] with Tg(Cd4-cre)1Cwi mice (Jackson Laboratories, Bar Habour, Main, USA - Stock No: 017336). Platelet CD40L-deficient mice (GP1baCre x CD40L fl/fl or GP1aCre) were generated by crossbreeding CD40L fl/fl mice [16] with GP1ba-Cre (platelet) mice [18]. A total of 6–10 mice were used per group.

Cre WT littermates served as a control for the respective cell-specific knockout model. Male cell-specific knockout mice and respective control mice (9–10 weeks) were bred in-house and treated with angiotensin-II (AT-II; 1 mg/kg/d in 0.9 % NaCl) by osmotic minipumps (Alzet model 1007D, Cupertino, CA, USA) for seven days. The pump implantation and tissue harvesting follow the same procedure described for the C57BL/6J mice.

### 2.2 Transthoracic echocardiography

High-frequency small-animal echocardiography was performed using a 38 MHz linear array transducer and Vevo3100 high-resolution imaging system (VisualSonics, Fujifilm, Toronto, Canada). The mice were anesthetized with 1.5 vol % isoflurane under permanent monitoring of ECG, breathing rate, and body temperature. The body temperature of the mice was kept constant via a heating system within the handling platform and infrared warming lamps. The parasternal long axis (PLAX) and parasternal short axis (SAX) were acquired in brightness (B)-mode and motion (M)-mode movies. The analysis was performed with the VevoLab Software (VisualSonics, Fujifilm, Toronto, Canada) [[19], [20], [21]]. The echocardiography was performed on day six of the treatment.

### 2.3 Non-invasive blood pressure measurements

Non-invasive blood pressure (bp) measurements were performed by plethysmography using the CODA system (Kent Scientific Corporation, Torrington, CT, USA) [11,22,23]. The mice were trained three times before measuring blood pressure to minimize stress reactions. Before the measurements, the animals freely entered the restraining tube, and the tubes were placed on a preheated platform for at least 15 min. The blood pressure was recorded on day five after the beginning of the treatment. Ten measurements were taken per animal; the first three were not included in the evaluation and were removed as acclimation cycles. The final data points reflect a mean value of seven independent measurements. The measurements were performed simultaneously to exclude diurnal variation of the blood pressure.

### 2.4 Vascular isometric tension studies

The endothelial function of aortic rings was measured via isometric tension studies as previously described [4,22,23]. Fat-free thoracic aortic rings of 3 mm length were mounted in organ bath chambers to force transducers (Kent Scientific Corporation, Torrington, CT, USA; Powerlab, ADInstruments, Spechbach, Germany) for the measurement. Pre-constriction of the aortic rings was performed with prostaglandin F_2α_, and endothelium-dependent relaxation was measured by adding different acetylcholine concentrations (Ach 10^−9^ – 10^−5.5^).

### 2.5 Detection of oxidative stress in different tissues

As described previously, the whole-blood oxidative burst was measured in fresh citrate blood [15,24]. The blood was mixed 1:50 in PBS and stimulated with either 50 μg/ml Zymosan A or 10 μM Phorbol 12,13-dibutyrate (PDBu). The chemiluminescence was measured using a Mithras^2^ plate reader (Berthold Technologies, Bad Wildbad, Germany). The results are expressed as counts per minute after 10 min (PDBu) or after 55 min (Zymosan A) and normalized to respective white blood cell count in the whole blood sample (Sysmex, Kobe, Japan).

The following tissues were used for the dihydroethidium (DHE) staining: aortic segments of 3 mm with perivascular fat, left ventricles, and kidney poles, as previously described [22,25]. The tissues were embedded in Tissue-Tek O.C.T. compound-resin (optimal Cutting Temperature, Sakura, Japan), frozen in liquid nitrogen, and cut into 8 μM cryosections. This was followed by an incubation with dihydroethidium (DHE; 1 μM in PBS) for 30 min at 37 °C in the dark. The fluorescence was detected using a Zeiss Axiovert 40 CFL microscope with a rhodamine filter and a DAPI filter. Fluorescence images were quantified as integrated optical density (IOD) using Image J software (NIH, MD, USA).

Superoxide anion detection by lucigenin-derived chemiluminescence was performed as previously described [5,26]. Fat-free thoracic aortic rings of 3 mm and 5 μM lucigenin were utilized for the counter measurement (Lumat LB 9507, Berthold Technologies, Bad Wildbad, Germany). The chemiluminescence value was normalized to the dry weight of the aortic rings.

### 2.6 Flow cytometry analysis of aortic lysates

The fat-free aortas were mechanically minced and digested with liberase TM (1 mg/ml, Roche Diagnostic, Basel, Swiss) for 30 min at 37 °C as previously described [19,27]. The cell suspension was transferred through a 70 μm cell strainer. And the cells were counted. Unspecific bindings were blocked with Fc-block (anti-CD16/CD32). The single cell suspensions were stained with the following monoclonal antibodies: PE anti-CD11b clone M1/70 (BD Bioscience, Franklin Lakes, NJ, USA), APC-eFluor™ 780 anti-CD45 clone 30-F11 (eBioscience, San Diego, CA, USA), APC anti-F4/80 clone BM8 (eBioscience, San Diego, CA, USA), PerCP-Cy^TM^5.5 anti-Ly6C clone AL-21 (BD Bioscience, Franklin Lakes, NJ, USA), FITC anti-Ly6G clone 1A8 (BD Bioscience, Franklin Lakes, NJ, USA), PE-Cyanine7 anti-NK1.1 clone PK136 (eBioscience, San Diego, CA, USA), V450 anti-TCRβ chain clone H57-597 (BD Bioscience, Franklin Lakes, NJ, USA), and eFluor™ 506 fixable viability dye (BD Bioscience, Franklin Lakes, NJ, USA). Events were recorded using an Attune NxT Flow Cytometer (Thermo Fisher, Waltham, MA, USA) and FlowJo software version 10 (Treestar, Ashland, Oregon, USA) for the data evaluation. The living cell count was normalized to the length of the respective aorta. The figure containing the flow cytometry data (see below) and Supplement Fig. 1 show representative plots and the gating strategy.

### 2.7 Statistics

Data are presented as means ± standard derivation (SD) if not stated. Statistical analysis was performed with GraphPad Prism software, version 9.5.1 (Graph Pad Software Inc., La Jolla, CA, USA). Outliers were identified and removed based on the ROUT (Q = 1 %) method. The Sapiro-Wilk test was used as a normality test. If the normality test passed, one-way ANOVA with Tukey's multiple comparison or two-way ANOVA with Dunnett's multiple comparison test, depending on the group design, were performed. If the normality test failed, the Kruskal-Wallis test with Dunn's multiple comparison test was performed. P-values <0.05 were considered statistically significant and labeled as ∗p ≤ 0.05, ∗∗p ≤ 0.01, ∗∗∗p ≤ 0.001, and ∗∗∗∗p ≤ 0.0001.

## 3 Results

### 3.1 TRAF6 inhibition in hypertensive mice decreases oxidative stress and improves endothelial function

**Fig. 1:**
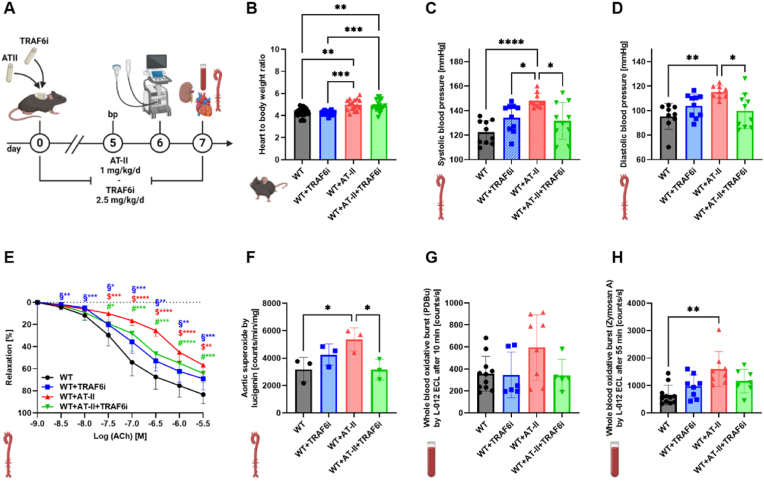
**CD40-TRAF6 inhibition leads to phenotypic improvement in hypertensive mice.** (A) WT animals were treated with either TRAF6i (2.5 mg/kg/d), AT-II (1 mg/kg/d), or AT-II and TRAF6i via osmotic minipumps for seven days. The blood pressure was measured on day five, echocardiography was performed on day six, and the organs were harvested on day seven. (B) At the end of the treatment, the heart and body weight were measured to calculate the heart-to-body weight ratio. (C, D) The systolic blood pressure was determined on day six of the treatment via non-invasive tail-cuff blood pressure measurement. The systolic blood pressure data were reused from our previously published work [11] with permission, whereas diastolic blood pressure data were not shown before. (E) Endothelial function in response to acetylcholine was measured via isometric tension studies. (F) Aortic O_2_^−•^ production was analyzed by using the chemiluminescence probe lucigenin. (G, H) The chemiluminescence of L-012 oxidation was utilized to analyze the mainly leukocyte-dependent H_2_O_2_ production in whole blood samples. The blood was stimulated with PDBu for 10 min (G) or zymosan A for 55 min (H). Data are presented as mean values ± SD of n = 15–16 (B), n = 9–10 (C, D), n = 6–16 (E), n = 3 (F) and n = 5–12 (G, H) animals per group. ∗p ≤ 0.05, ∗∗p ≤ 0.01, and ∗∗∗p ≤ 0.001. One-way ANOVA with Tukey's multiple comparison test (B, C, D, F, G, H) and two-way ANOVA with Dunnett's multiple comparison test (E) were performed. (A) and all icons were created with BioRender.com. Abbreviations: WT=C57BL6/J mice, TRAF6i = TRAF6 inhibitor, AT-II = angiotensin-II, bp = blood pressure, Ach = acetylcholine, PDBu = phorbol 12,13-dibutyrate.

**Fig. 2:**
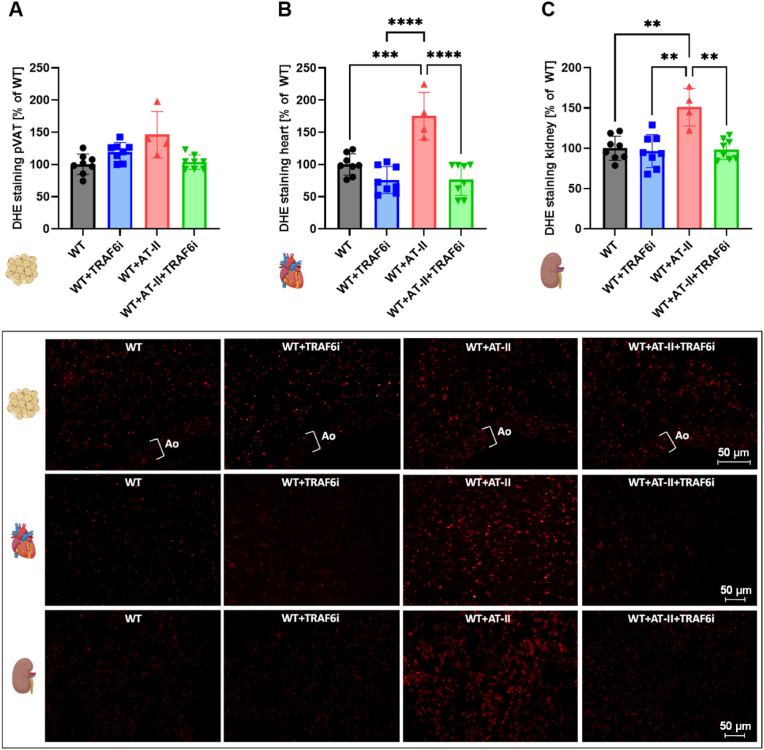
**CD40-TRAF6 inhibition leads to reduced ROS formation in different tissues of hypertensive mice.** The overall ROS production was analyzed in cryosections via DHE staining in different tissues: pVAT (A), heart (B), and kidney (C). Representative DHE pictures are shown below the bar graphs. The red color reflects the fluorescence from O_2_^−•^ and H_2_O_2_. Data are presented as mean values ± SD of n = 4–8 (A–C) animals per group. ∗p ≤ 0.05, ∗∗p ≤ 0.01, ∗∗∗p ≤ 0.001, and ∗∗∗∗p ≤ 0.0001. One-way ANOVA with Tukey's multiple comparison test was performed. Icons were created with BioRender.com. Abbreviations: WT=C57BL6/J mice, TRAF6i = TRAF6 inhibitor, AT-II = angiotensin-II, DHE = dihydroethidium, pVAT = perivascular adipose tissue, Ao = aorta.

Arterial hypertension in WT mice (C57BL/6J) was induced via AT-II treatment for seven days. In addition, the mice were treated with TRAF6 inhibitor compound 6877002 to diminish the inflammatory phenotype. An overview of the experimental protocol is shown in Fig. 1 A. The heart-to-body weight ratio was not significantly reduced in hypertensive animals after TRAF6 inhibitor treatment (Fig. 1 B). Meanwhile, the systolic and diastolic blood pressure was increased in AT–II–infused mice and significantly decreased by TRAF6 inhibition in hypertensive animals (Fig. 1C and D). TRAF6 inhibition also significantly improved the endothelial dysfunction caused by hypertension (Fig. 1 E). Aortic superoxide production measured by lucigenin was reduced considerably in hypertensive mice with TRAF6 inhibitor treatment (Fig. 1 F). No significant differences were observed after PDBu stimulation in whole blood oxidative burst measurements (Fig. 1 G). In zymosan A stimulated whole blood samples, the oxidative burst was significantly increased in AT-II treated mice compared to the WT control but only slightly decreased in hypertensive TRAF6 inhibitor-treated mice (Fig. 1H). ROS formation was analyzed in cryosections of perivascular adipose tissue (pVAT) (Fig. 2 A), cardiac (Fig. 2 B), and renal (Fig. 2C) tissue via DHE staining. In cardiac and renal tissue of hypertensive mice, ROS were significantly reduced after TRAF6 inhibitor treatment.

**Fig. 3:**
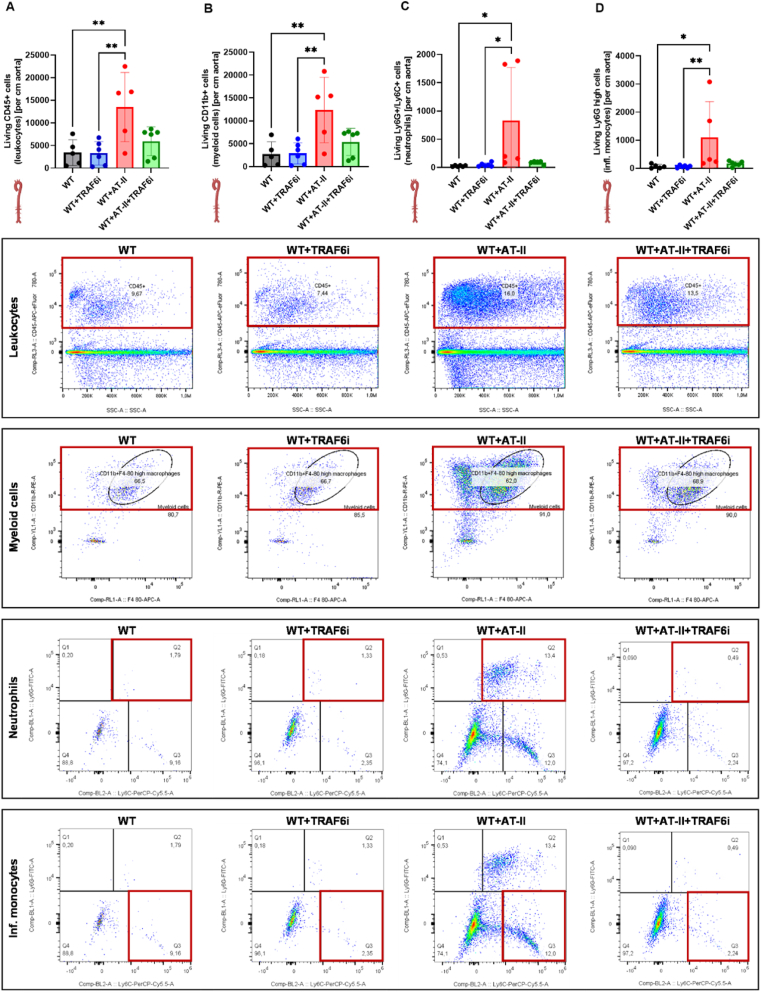
**CD40-TRAF6 inhibition reduces vascular immune cell infiltration in hypertensive mice.** The immune cell content in aortic tissue lysates was analyzed via flow cytometry with different fluorophore-tagged antibodies. The following antibodies were used to distinguish between different immune cell subtypes: (A) anti-CD45 (leukocytes), (B) anti-CD11b (myeloid cells), (C + D) anti-Ly6G and anti-Ly6C (inflammatory monocytes and neutrophils). Representative counter blots are shown below the bar graphs, and the area of interest is marked with a red box. Data are presented as mean values ± SD of n = 5–6 (A–D) animals per group. ∗p ≤ 0.05 and ∗∗p ≤ 0.01. One-way ANOVA with Tukey's multiple comparison test was performed. Icons were created with BioRender.com. Abbreviations: WT=C57BL6/J mice, TRAF6i = TRAF6 inhibitor, AT-II = angiotensin-II, CD = cluster of differentiation, Infl = inflammatory.

### 3.2 TRAF6 inhibition minimizes vascular immune cell infiltration in hypertensive mice

Vascular inflammation of hypertensive mice was reflected through high leukocyte (CD45^+^; Fig. 3 A), myeloid (CD11b^+^, Fig. 3 B), neutrophil (Ly6G^+^/Ly6C^+^; Fig. 3C), and inflammatory monocyte (Ly6C high; Fig. 3 D) cell counts in flow cytometry measurements of aortic tissue lysates. CD45^+^ cells were increased in the WT + AT-II group compared to the control groups. TRAF6 inhibitor treatment in hypertensive animals resulted in reduced total CD45^+^ leukocyte (p = 0.0504), CD11b^+^ myeloid (p = 0.0534), Ly6C^high^ monocyte, and neutrophils (p = 0.0685) cell counts. Representative counterplots are shown below the bar graphs, and the area of interest is marked in red.

### 3.3 TRAF6 inhibition does not prevent left ventricular hypertrophy in hypertensive mice

**Fig. 4:**
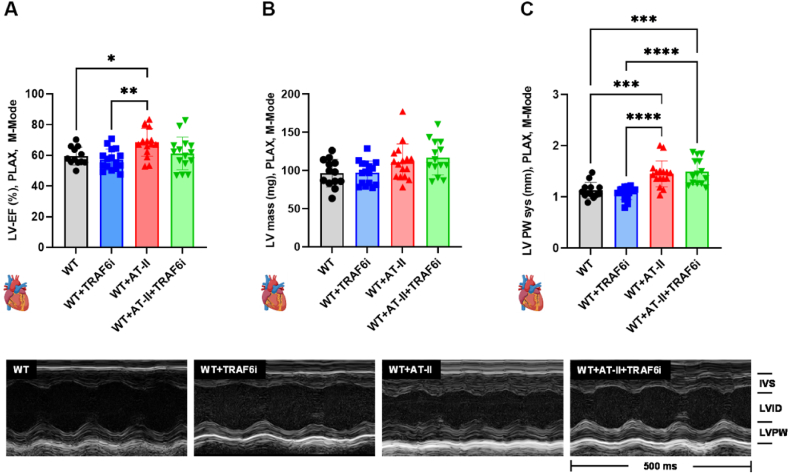
**CD40-TRAF6 inhibition does not reduce left ventricular hypertrophy in hypertensive mice.** After six days of treatment, transthoracic echocardiography for small animals was performed to determine left ventricular (LV) function. The Vevo Lab 3100 software was used to calculate the following parameters: (A) LV-EF, (B) LV mass, and (C) LV PW (systolic). Representative M-mode pictures are shown below the bar graphs. Data are presented as mean values ± SD of n = 12–16 (A–C) animals per group. ∗∗p ≤ 0.05, ∗∗p ≤ 0.01, ∗∗∗p ≤ 0.001, and ∗∗∗∗p ≤ 0.0001. One-way ANOVA with Tukey's multiple comparison test was performed. Icons were created with BioRender.com. Abbreviations: WT=C57BL6/J mice, TRAF6i = TRAF6 inhibitor, AT-II = angiotensin-II, LV = left ventricle, EF = ejection fraction, PLAX = parasternal long axis view, M-mode = motion mode, PW = posterior wall, sys = systole, IVS = intraventricular septum, LVID = left ventricular internal diameter, LVPW = left ventricular posterior wall.

Left ventricular function measurements were performed using transthoracic small animal echocardiography. The left ventricular ejection fraction (LV-EF) was significantly increased in hypertensive mice compared to WT and WT + TRAF6i mice. WT + AT-II + TRAF6i mice did not differ substantially from the other treatment groups (Fig. 4 A). LV mass was slightly increased in the WT + AT-II and WT + AT-II + TRAF6i groups (Fig. 4 B). The LV posterior wall (LVPW) was significantly thicker in the AT-II treated animals than in the control groups. TRAF6 inhibitor treatment did not improve the wall thickening in hypertensive mice (Fig. 4C). Representative M-Mode pictures are shown below the bar graphs.

### 3.4 Adipocyte, B-cell, and T-cell-specific CD40 or platelet-specific CD40L knockout does not improve endothelial function or reduce systolic blood pressure in hypertensive mice

**Fig. 5:**
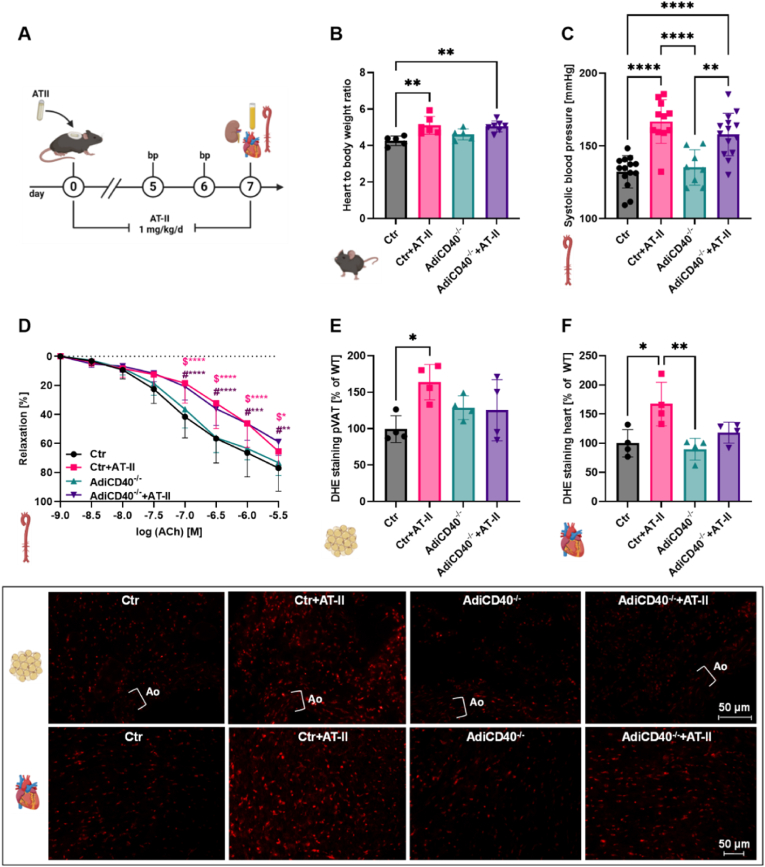
**Adipocyte-specific CD40 knockout shows no vasoprotective phenotype in hypertensive mice.** (A) Adipocyte-specific CD40^−/−^ (AdiCD40^−/−^) and respective control mice (Ctr) were treated for seven days with AT-II (1 mg/kg/d) via osmotic minipumps. The blood pressure was measured on days five and six. The organs were harvested on day seven. (B) At the end of the treatment, the heart and body weight were measured to calculate the heart-to-body weight ratio. (C) The systolic blood pressure of the mice was determined via non-invasive tail-cuff blood pressure measurement. (D) Endothelial function in response to acetylcholine was measured via isometric tension studies. The ROS production was analyzed in cryosections via DHE staining in pVAT (E) and cardiac (F) tissue. Representative DHE pictures are shown below the bar graphs. Data are presented as mean values ± SD of n = 5–7 (B), n = 8–14 (C), n = 9–14 (D), and n = 4 (E, F) animals per group. ∗p ≤ 0.05, ∗∗p ≤ 0.01, ∗∗∗p ≤ 0.001, and ∗∗∗∗p ≤ 0.0001. One-way ANOVA with Tukey's multiple comparison test (B, C, E, F) and two-way ANOVA with Dunnett's multiple comparison test (D) were performed. Icons were created with BioRender.com. Abbreviations: Ctr = AdiCD40^−/−^ (Cre WT), TRAF6i = TRAF6 inhibitor, AT-II = angiotensin-II, bp = blood pressure, DHE = dihydroethidium, pVAT = perivascular adipose tissue, ACh = acetylcholine.

CD40-expressing adipocytes are essential in modulating immune cell responses, especially in cardiovascular and metabolic disease progression. The AdiCD40^−/−^ mouse model was utilized to study the role of adipocytes in CD40L-CD40-mediated inflammatory signaling. AdiCD40^−/−^ and respective control mice were treated for seven days with 1 mg/kg/d AT-II to induce arterial hypertension (Fig. 5 A). A significant increase in the heart-to-body weight ratio and systolic blood pressure was observed in the AT-II treated Ctr and AdiCD40^−/−^ mice compared to the respective untreated mice. The adipocyte-specific CD40 KO did not improve cardiac hypertrophy and systolic blood pressure in hypertensive animals (Fig. 5B and C). The endothelial function was measured via isometric tension studies and significantly impaired in Ctr and AdiCD40^−/−^ AT-II treated mice. However, no significant differences between the two AT-II treated groups were observed, indicating that adipocyte-specific CD40 KO did not improve endothelial function (Fig. 5 D). The overall ROS production in pVAT and cardiac tissue was significantly increased in the Ctr AT-II group compared to control animals. The ROS production in the hypertensive adipocyte-specific CD40^−/−^ mice was reduced by trend but not significantly compared to the Ctr + AT-II group (Fig. 5E and F). Representative pictures of cardiac tissue are shown below the bar graphs, where the red indicates the DHE staining.

**Fig. 6:**
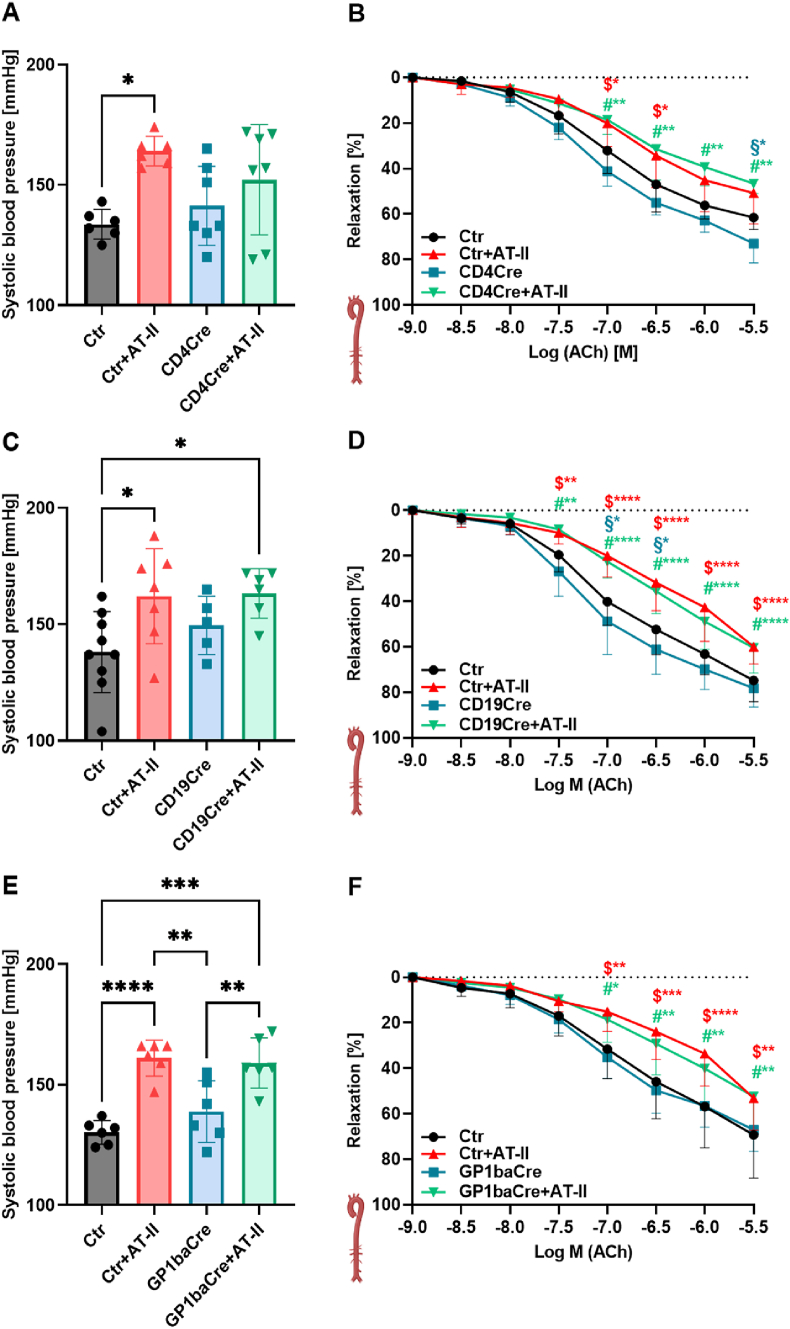
**Tissue-specific CD40/CD40L shows no vasoprotective phenotype in hypertensive mice.** B-cell specific (CD4Cre) CD40 knockout mice, T-cell specific (CD19Cre) CD40 knockout mice, and platelet (GP1baCre) CD40L specific knockout mice and respective control mice (Ctr) were treated for seven days with AT-II (1 mg/kg/d) via osmotic minipumps. (A,C,E) The systolic blood pressure was determined on day six of the treatment via non-invasive tail-cuff blood pressure measurement. (B,D,F) Endothelial function in response to acetylcholine was measured via isometric tension studies. Data are presented as mean values ± SD of n = 6–7 (A-), n = 6–9 (B), n = 6–9 (C), n = 10–13 (D), n = 6 (E), n = 7–11 (F) animals per group. ∗∗p ≤ 0.05, ∗∗p ≤ 0.01, ∗∗∗p ≤ 0.001, and ∗∗∗∗p ≤ 0.0001. One-way ANOVA with Tukey's multiple comparison test (A,C,E) and two-way ANOVA with Dunnett's multiple comparison test were performed (B,D,F). Icons were created with BioRender.com. Abbreviations: Ctr = CD4Cre od CD19Cre or GP1baCre (Cre WT), AT-II = angiotensin-II, ACh = acetylcholine.

In addition, neither CD4- and CD19-specific CD40 deficiency (accounting for T cells and B cells) nor GPb1a-specific CD40L deficiency (accounting for platelets) showed improvement in blood pressure or endothelial dysfunction (Fig. 6). Also, aortic and cardiac ROS formation, as measured by DHE staining, was not improved by any of the investigated cell-specific CD40 or CD40L deficiencies (Suppl. Figs. S2–S4). In contrast, whole blood oxidative burst as a read-out of leukocyte-derived ROS formation was suppressed by CD4- and CD19-specific CD40 knockout, whereas GPb1a-specific CD40L knockout had no effects on the oxidative burst in AT-II treated mice (Suppl. Figs. S2–S4).

## 4 Discussion

Results of the present studies establish a protective effect by employing a CD40-TRAF6 inhibitor in an animal model for arterial hypertension. In addition to the reduction in systolic blood pressure by CD40-TRAF6 inhibition, as we described before [11], we further present evidence of improved endothelial function, reduced oxidative stress and diminished aortic immune cell infiltration in hypertensive mice with CD40-TRAF6 signaling blockade. While TRAF6 inhibition was effective in a hypertensive mouse model, cell type-specific CD40 deficiency in adipocytes, T-cells, and B-cells or CD40L deficiency in platelets did not show protective effects.

### 4.1 Effects of CD40-TRAF6 inhibition

CD40 signaling acts as a double-edged sword since CD40 has two binding sites one for TRAF2/3/5 and one for TRAF6 [3]. Previously, it was shown that CD40-TRAF2/3/5 signaling protects against metabolic dysfunction and inflammation in a mouse model of a metabolic syndrome. In contrast, the CD40-TRAF6 pathway contributes to the harmful consequences of obesity. In the same mouse model, selective CD40-TRAF6 inhibition improved insulin resistance and adipose tissue inflammation [6]. Additionally, CD40-TRAF6 inhibition in Apoe^−/−^ mice reduced atherosclerosis in young animals and stopped plaque progression in older mice. CD40-associated immunity, such as T cell proliferation, Ig isotype switching, or germinal center formation, was not impaired by CD40-TRAF6 inhibition [10]. In contrast to a global CD40 depletion, selective CD40-TRAF6 inhibition preserves CD40-associated immunity mediated by TRAF2/3/5 signaling. The here-presented protective effects of CD40-TRAF6 inhibition align with our previous findings in db/db mice, where the same compound improved endothelial dysfunction, reduced ROS formation, and decreased cardiac expression of inflammatory markers [4]. Flow cytometric analysis shows that TRAF6 inhibition reduces the massive invasion of leukocytes into the aortic wall of hypertensive mice. Additionally, ROS production was suppressed in hypertensive mice by TRAF6i therapy as indicated by reduced DHE staining and oxidative burst measurement in the aorta, heart, kidney, and whole blood. This further strengthens the postulated concept of the immune system being an effector in developing arterial hypertension [28]. The lack of reduction in LV mass and concentric cardiac remodeling and an increased heart-to-body weight ratio in hypertensive TRAF6 inhibitor-treated animals can be explained by blood pressure-independent effects of AT-II on heart muscle as first described by Mazzolai and colleagues [29]. In vitro studies conducted by Cao and colleagues found a TRAF6-dependent reaction to AT-II, which involves the TGF-β pathway and results in hypertrophied cardiomyocytes [30], which do not have specific clinical effects.

Angiotensin-II infusion in mice induces a thromboinflammatory phenotype promoting infiltration of immune cells into cardiovascular tissues and CD40L- [5], interferon-gamma- [31] or tissue factor-dependent immune cell activation [15,32]. The phagocytic NADPH oxidase, with NOX2 and p47phox as key components, plays a key role in developing hypertension in this model [[12], [13], [14], [15]]. In particular, the influx of immune cells into the aortic wall represents a key pathomechanisms of blood pressure dysregulation as it is also observed in animals subjected to noise [33], DOCA salt [34,35] or particulate matter [36]. Previous work has also shown that AT-II infusion caused substantial impairment of endothelial nitric oxide synthase (eNOS) function by oxidative degradation of the essential cofactor tetrahydrobiopterin (BH4) to dihydrobiopterin [37] or adverse redox signaling via eNOS uncoupling by S-glutathionylation of a critical cysteine thiol group in the reductase domain [24]. Depletion of BH4 by peroxynitrite or superoxide/hydrogen peroxide as well as oxidative stress-mediated S-glutathonlyation of eNOS represent accepted mechanisms of eNOS uncoupling [[38], [39], [40]]. Accordingly, the improved endothelial function by TRAF6 inhibition is the suppression of AT–II–induced oxidative stress and prevention of oxidative eNOS uncoupling, as reviewed in detail previously [41]. Also, targeted cardiac overexpression of angiotensin-converting enzymes resulted in cardiac oxidative stress, eNOS uncoupling, and diastolic dysfunction, complications that were improved by oral administration of BH4 [42]. In general, endothelial function is tightly connected to AT-II signaling [43].

### 4.2 Effects of adipocyte, B-cell, and T-cell-specific CD40 or platelet-specific CD40L deficiency

CD40-expressing adipocytes modulate immune cell activation and play a key role in inflammation [44,45]. In our study, the hypertensive phenotype in mice was not improved by selective adipocyte CD40 depletion, demonstrated by endothelial function and oxidative stress measurements in aortic and cardiac tissue. A similar adipocyte-specific CD40 knockout mouse model was used in a recently published study, and it was shown that adipocytes control hematopoiesis and inflammation via CD40 signaling. Further, the role of adipocyte-specific knockout mice in atherosclerosis was investigated by backcrossing the mice to ApoE^−/−^ mice and fed with a high-cholesterol diet. Also, in this model, the systemic immune cell composition was affected, whereas the body weight, plasma lipid levels, and triglycerides were not significantly changed. The atherosclerotic lesion size was reduced in this mouse model, but the necrotic core formation was induced. Lastly, in obese adipocyte-specific deficient mice, reduced weight gain, fewer immune cells in adipose tissue, and increased fat oxidation were observed [17]. We consider CD40-expressing adipocytes to be more involved in the progression of atherosclerosis and obesity than in the development of arterial hypertension. Further, our study did not focus on the cellular regulation of the immune system in the context of hypertension. Opposite to our findings, CD40 deficiency on follicular B cells was described as protective in LDL receptor-deficient mice, which suffer from severe hypercholesterolemia [46]. CD40^+^ B-cells have also been associated with a higher risk of stroke in the future [47]. Even though B-cells have been shown to play various roles in the development of atherosclerosis, both beneficial and detrimental [48], our data suggest that in arterial hypertension, blocking CD40 on B-cells or T-cells is not beneficial. Also, no beneficial effects on endothelial function or oxidative stress were observed in hypertensive mice due to platelet-specific CD40L depletion. In hypertensive global CD40L knockout animals, protective effects like improved endothelial dysfunction, reduced platelet monocyte interaction in blood, and decreased oxidative stress in the cardiovascular system were demonstrated [5].

In summary, we conclude that adipocytes, B-cells, and T-cells are not the main drivers of CD40L-CD40 mediated inflammation regarding the hypertensive mouse models utilized for this study. We suggest focusing more on macrophage-driven inflammation in the future. Recently, it was shown that mice with a myeloid-specific CD40 knockout on an ApoE^−/−^ background developed a less activated immune cell profile, reduced atherosclerosis, and increased alternative macrophage activation [49]. Also, endothelial-specific CD40 deficiency in an atherosclerotic mouse model is associated with a more stable plaque phenotype and decreased leukocyte adhesion. In humans, endothelial CD40 expression is also increased in atherosclerotic patients compared to controls [7]. However, it is hard to predict whether CD40-TRAF6 inhibition on endothelial cells can improve endothelial function or reduce hypertension. At least, previous work has shown that treatment of cultured endothelial cells or isolated mouse aorta with CD40L causes oxidative damage of ECs, e.g. by nitration and inactivation of prostacyclin synthase by enhanced peroxynitrite formation, and induction of ICAM-1 expression [50]. Removal of CD40 by siRNA, adenoviral overexpression of superoxide dismutase or pharmacological inhibition of eNOS in cultured ECs prevented the CD40L-dependent detrimental effects, suggesting enhanced superoxide formation as a key mechanism in this pathophysiological process. Accordingly, one may speculate that inhibition of CD40-TRAF6 signaling in ECs may be cardioprotective and antihypertensive.

## 5 Limitations of the study

Hypertension in humans is multifactorial, with the renin-angiotensin-aldosterone system playing a significant role. Despite this, we believe this model is appropriate for our studies, particularly regarding CD40L–CD40–TRAF signaling. Our earlier findings indicated that the AT-II model primarily involves recruiting inflammatory cells that express CD40L. The adverse effects of AT–II–induced arterial hypertension on vascular function and significant blood pressure increases are well-documented and have been extensively studied, particularly by our research group and others [15,34].

Although the AT–II–induced hypertension models in mice or rats can lead to severe hypertension, heightened oxidative stress, and inflammation, they are commonly used to investigate the pathophysiology of arterial hypertension over a short duration of 1–2 weeks. The effectiveness of traditional antihypertensive drugs, such as AT1 receptor blockers and ACE inhibitors, further underscores the relevance of these models to human hypertension. Our mouse model of arterial hypertension induced by AT-II treatment represents just one potential pathway contributing to the development of hypertension. Furthermore, the one-week AT-II infusion model does not capture the full extent of end-organ damage caused by hypertension, such as remodeling and oxidative modifications in the kidney and heart. As a result, we could not thoroughly assess the effects of TRAF6 inhibition on end-organ damage in this study. However, we have previously demonstrated that global CD40L deficiency can prevent all significant complications associated with AT-II treatment [5].

We have previously demonstrated that the AT-II model is primarily driven by the influx of inflammatory cells expressing CD40L. The detrimental effects of AT–II–induced arterial hypertension on vascular function and significant increases in blood pressure are well-documented and have been extensively studied, particularly by our research group [5,51]. While the AT–II–induced hypertension models in mice and rats can lead to severe hypertension and notable increases in oxidative stress and inflammation, they are commonly used to investigate the pathophysiology of arterial hypertension over a short duration of 1–2 weeks. The effectiveness of traditional antihypertensive medications, such as AT1 receptor blockers and angiotensin-converting enzyme inhibitors, further underscores the relevance of these models to human hypertension.

## 6 Conclusion and clinical impact

In hypertensive mice, the adipocyte, T-cell, and B-cell-specific CD40 depletion or platelet-specific CD40L depletion demonstrated a minor effect on endothelial function and oxidative stress in aortic and cardiac tissue. We conclude that these cell types are not the main drivers of CD40L-CD40-mediated vascular inflammation in the context of hypertension. From the evidence in the literature, we suggest focusing more on macrophage-driven inflammation in the future regarding hypertension. Meanwhile, TRAF6 inhibitor treatment in hypertensive animals showed beneficial effects such as improved endothelial dysfunction, reduced oxidative stress in cardiovascular tissues, and reduced aortic immune cell infiltration. We conclude that TRAF6 inhibition reduces pathophysiological vascular inflammation and oxidative damage in hypertensive mice and that TRAF6 inhibition could be a suitable candidate for a therapeutic approach in hypertensive patients.

## CRediT authorship contribution statement

**Lea Strohm:** Writing – review & editing, Writing – original draft, Methodology, Investigation, Formal analysis, Data curation, Conceptualization. **Henning Ubbens:** Writing – review & editing, Methodology, Investigation, Formal analysis. **Dominika Mihalikova:** Writing – review & editing, Methodology, Investigation. **Alexander Czarnowski:** Writing – review & editing, Methodology, Investigation. **Paul Stamm:** Writing – review & editing, Supervision, Methodology, Funding acquisition. **Michael Molitor:** Writing – review & editing, Methodology, Investigation, Formal analysis, Data curation. **Stefanie Finger:** Writing – review & editing, Methodology, Investigation, Formal analysis. **Matthias Oelze:** Writing – review & editing, Validation, Methodology, Formal analysis. **Dorothee Atzler:** Writing – review & editing, Project administration, Methodology. **Philip Wenzel:** Writing – review & editing, Supervision, Methodology, Funding acquisition. **Philipp Lurz:** Writing – review & editing, Project administration, Funding acquisition. **Thomas Münzel:** Writing – review & editing, Writing – original draft, Funding acquisition. **Christian Weber:** Writing – review & editing, Supervision, Resources, Methodology, Funding acquisition. **Esther Lutgens:** Writing – review & editing, Writing – original draft, Resources, Project administration, Methodology, Funding acquisition. **Andreas Daiber:** Writing – review & editing, Writing – original draft, Supervision, Conceptualization. **Steffen Daub:** Writing – review & editing, Writing – original draft, Supervision, Project administration, Methodology, Funding acquisition, Formal analysis, Conceptualization.

## Funding

SD was supported by a vascular biology research grant on “CD40L and inflammation in hypertension” of the Else-Kröner-Fresenius Foundation (2019_A110) and a research stipend from the foundation Heart of Mainz. LS and HU hold PhD stipends of the TransMed PhD Program financed by the Else-Kröner-Fresenius Foundation (2019_A110). TM is PI, and AD Scientist of the DZHK (German Center for Cardiovascular Research), Partner Site Rhine-Main, Mainz, Germany. This work was supported by 10.13039/501100001659German Research Foundation (DFG) grant of the Major Research Instrumentation Programme (DFG INST 371/47-1 FUGG). C.W. is a Van Der Laar professor of atherosclerosis.

## Declaration of competing interest

The authors declare the following financial interests/personal relationships which may be considered as potential competing interests:SD was supported by a vascular biology research grant on “CD40L and inflammation in hypertension” of the Else-Kröner-Fresenius Foundation (2019_A110) and a research stipend from the foundation Heart of Mainz. LS and HU hold PhD stipends of the TransMed PhD Program financed by the Else-Kröner-Fresenius Foundation (2019_A110). TM is PI, and AD Scientist of the DZHK (German Center for Cardiovascular Research), Partner Site Rhine-Main, Mainz, Germany. This work was supported by 10.13039/501100001659German Research Foundation (DFG) grant of the Major Research Instrumentation Programme (DFG INST 371/47-1 FUGG). C.W. is a Van Der Laar professor of atherosclerosis.

## Data Availability

All final data are available in the manuscript or the supplementary material. Raw data (source files) will be provided upon request. There is no final data missing in the published article.
