# Supplementary material for: CD40-TRAF6 inhibition suppresses cardiovascular inflammation, oxidative stress and functional complications in a mouse model of arterial hypertension
Source: Redox Biol. 2025 Jan 29;80:103520. doi: 10.1016/j.redox.2025.103520 (PMC11840497; doi:10.1016/j.redox.2025.103520)
Supplement: Multimedia component 1 [file mmc1.pdf]

Supplement

TRAF6 inhibition suppresses cardiovascular inflammation and reduces complications in a mouse model of arterial hypertension

Lea Strohm[1], Henning Ubbens[1], Dominika Mihalikova[1], Alexander Czarnowski[1], Paul Stamm[1], Michael Molitor[1,2,3], Stefanie Finger[2], Matthias Oelze[1], Dorothee Atzler[4,5,6], Philip Wenzel[1,2,3], Philipp Lurz[1], Thomas Münzel[1,2,3], Christian Weber[4,5,7], Esther Lutgens[5,8], Andreas Daiber[1,2,3#] and Steffen Daub[1]

[1] Department of Cardiology, Cardiology I, University Medical Center of the Johannes Gutenberg-University, Mainz, Germany; [2] Center for Thrombosis and Hemostasis, University Medical Center of the Johannes Gutenberg-University, Mainz, Germany; [3] German Center for Cardiovascular Research (DZHK), Partnersite Rhine-Main, Mainz, Germany; [4]Institute for Cardiovascular Prevention, Ludwig-Maximilians-Universität München, Munich, Germany; [5]DZHK (German Center for Cardiovascular Research), Partner Site Munich Heart Alliance, Germany; [6]Walter Straub Institute of Pharmacology and Toxicology, Ludwig-Maximilians-Universität, Munich, Germany; [7]Munich Cluster for Systems Neurology (SyNergy), Munich, Germany; [8] Mayo Clinic, Dept Cardiovascular Medicine and Immunology, Rochester, MN, USA.

Address correspondence to
Prof. Dr. Andreas Daiber, Universitätsmedizin der Johannes Gutenberg-Universität Zentrum für Kardiologie 1 – Labor für Molekulare Kardiologie, Geb. 605 – Raum 3.262, Langenbeckstr. 1, 55131 Mainz, Germany, Phone +49 (0)6131 176280, Fax +49 (0)6131 176293, E-mail: daiber@uni-mainz.de

Supplementary Figures

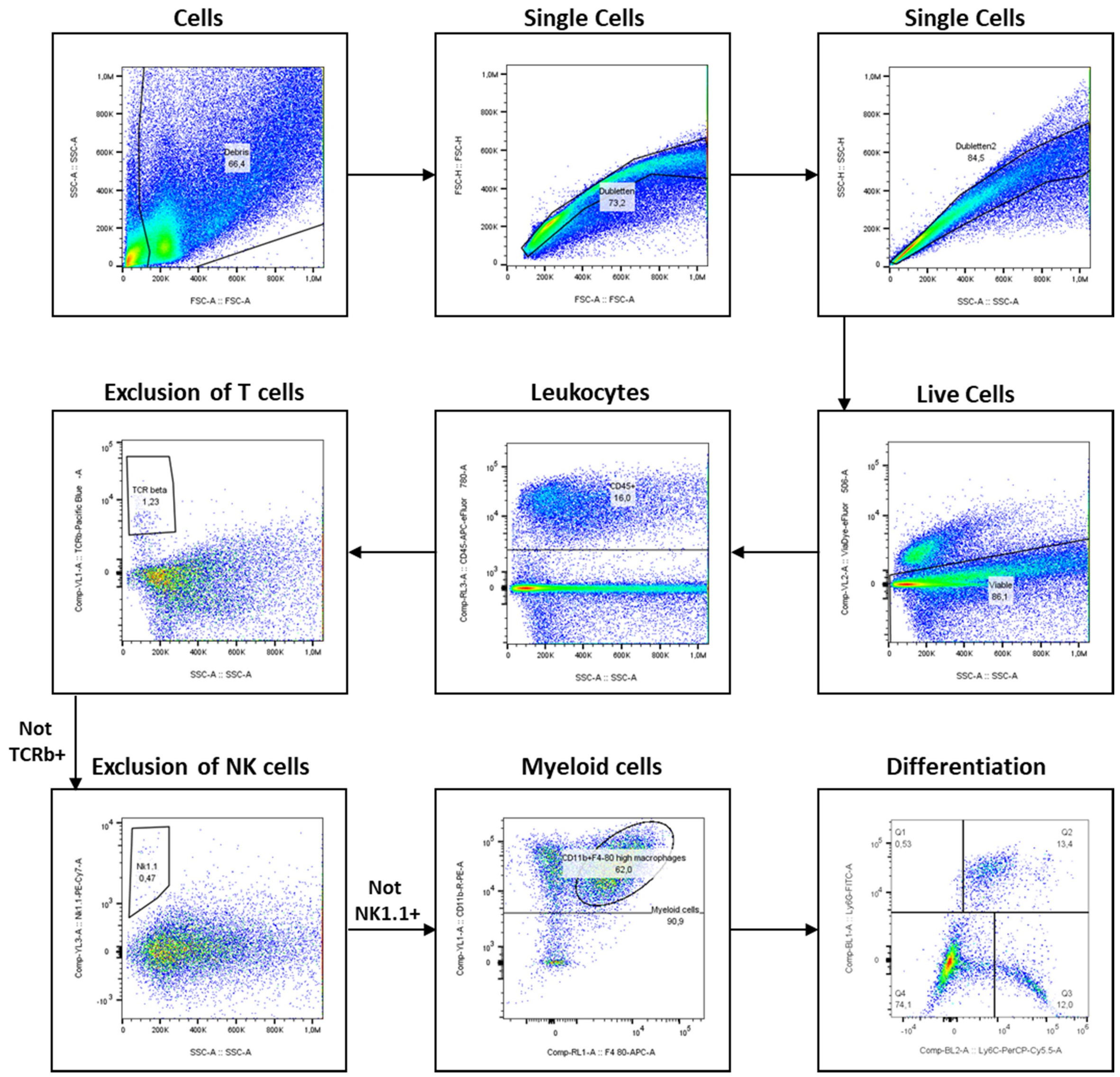

Supplement Figure 1: Gating strategy to identify immune cells in aortic single-cell suspensions via flow cytometry. Initially, debris (SSC-A against FSC-A), doublets (FSC-H against FSC-A and SSC-H against SSC-A), and dead cells (viability marker against SSC-A) were excluded. The gating of immune cells started with identifying CD45-positive leukocytes (CD45 marker against SSC-A). Followed by the identification and exclusion of T cells (TCRβ marker against SSC-A) and NK cells (NK1.1. marker against SSC-A). CD11b positive myeloid cells (CD11b marker against F4/80 marker) were identified and further differentiated into neutrophils (Ly6G and Ly6C positive) and inflammatory monocytes (Ly6C high).

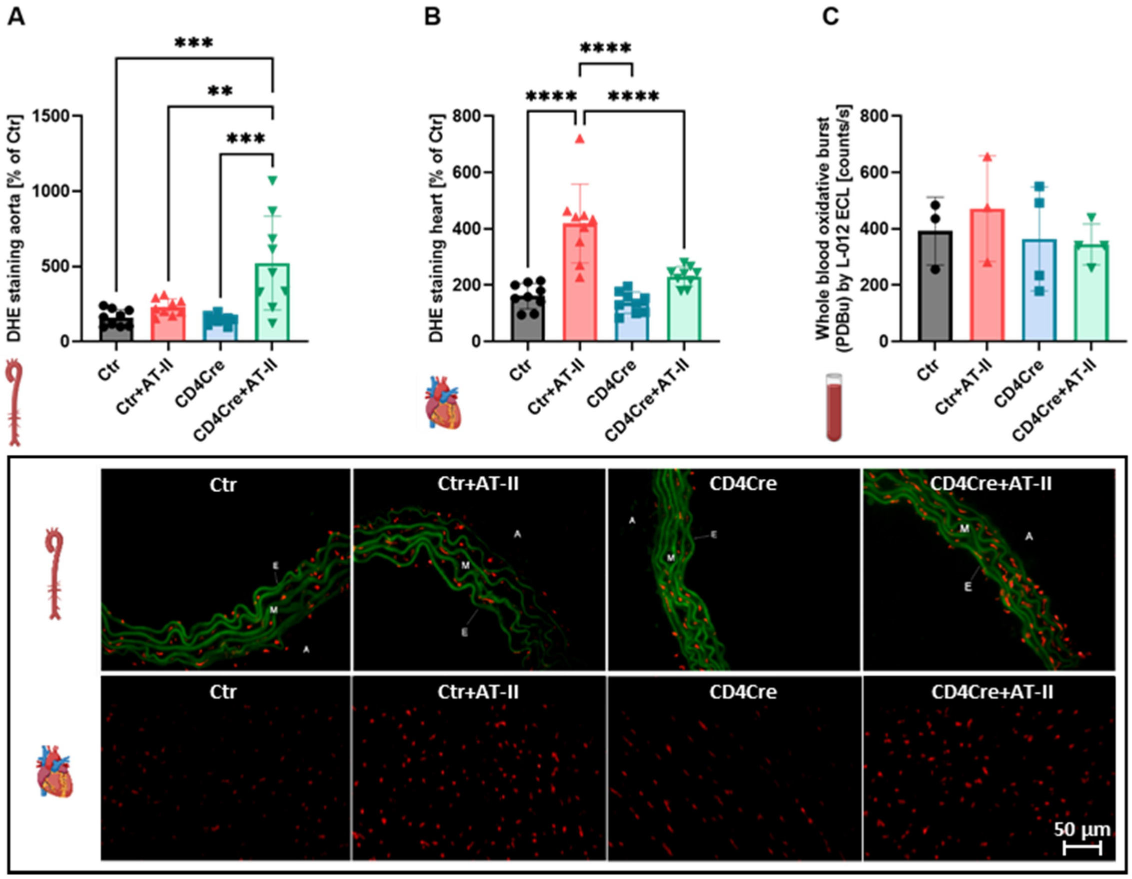

Supplement Figure 2: T-cell-specific CD40 knockout shows no protective effect against oxidative stress in hypertensive mice. T-cell-specific CD40 knockout mice (CD4Cre) and respective control mice (Ctr) were treated with AT-II (1mg/kg/d) for seven days using osmotic minipumps. After treatment, blood was collected, and organs were harvested. Total ROS production was analyzed in cryosections by DHE staining in different tissues: aorta (A) and heart (B). Representative DHE images are shown below the bar graphs. The chemiluminescence of L-012 oxidation was used to analyze the main leukocyte-dependent H_2O_2 production in whole blood samples. Blood was previously stimulated with PDBu. Data are presented as mean ± SD of n=9 (A-), n=9 (B), n=3-4 (C) animals. $p \leq 0.05$, **$p \leq 0.01$, ***$p \leq 0.001$ and ****$p \leq 0.0001$. One-way ANOVA with Tukey's multiple comparison test (A,B) and two-way ANOVA with Dunnett's multiple comparison test (C) were performed. Icons were created using BioRender.com. Abbreviations: AT-II=angiotensin II, PDBu=phorbol 12,13-dibutyrate.

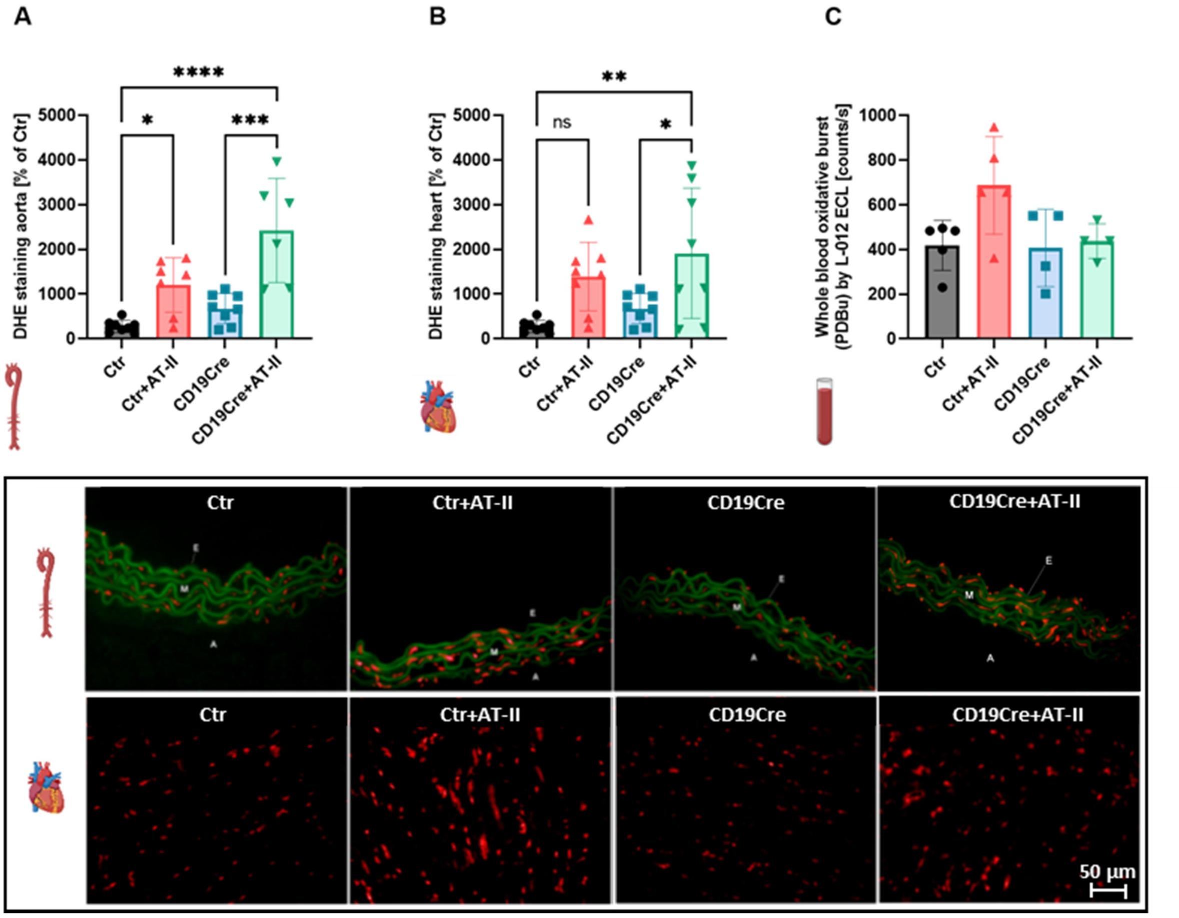

Supplement Figure 3: B-cell-specific CD40 knockout shows no protective effect against oxidative stress in hypertensive mice. B-cell-specific CD40 knockout mice (CD19Cre) and respective control mice (Ctr) were treated with AT-II (1mg/kg/d) for seven days using osmotic minipumps. After treatment, blood was collected, and organs were harvested. Total ROS production was analyzed in cryosections by DHE staining in different tissues: aorta (A) and heart (B). Representative DHE images are shown below the bar graphs. The chemiluminescence of L-012 oxidation was used to analyze the main leukocyte-dependent H_2O_2 production in whole blood samples. Blood was previously stimulated with PDBu. Data are presented as mean ± SD of n=24(A), n=24 (B), n=4-5 (C) animals. $p \leq 0.05$, $^{**}p \leq 0.01$, $^{***}p \leq 0.001$ and $^{****}p \leq 0.0001$. One-way ANOVA with Tukey's multiple comparison test (A,B) and two-way ANOVA with Dunnett's multiple comparison test (C) were performed. Icons were created using BioRender.com. Abbreviations: AT-II=angiotensin II, PDBu=phorbol 12,13-dibutyrate.

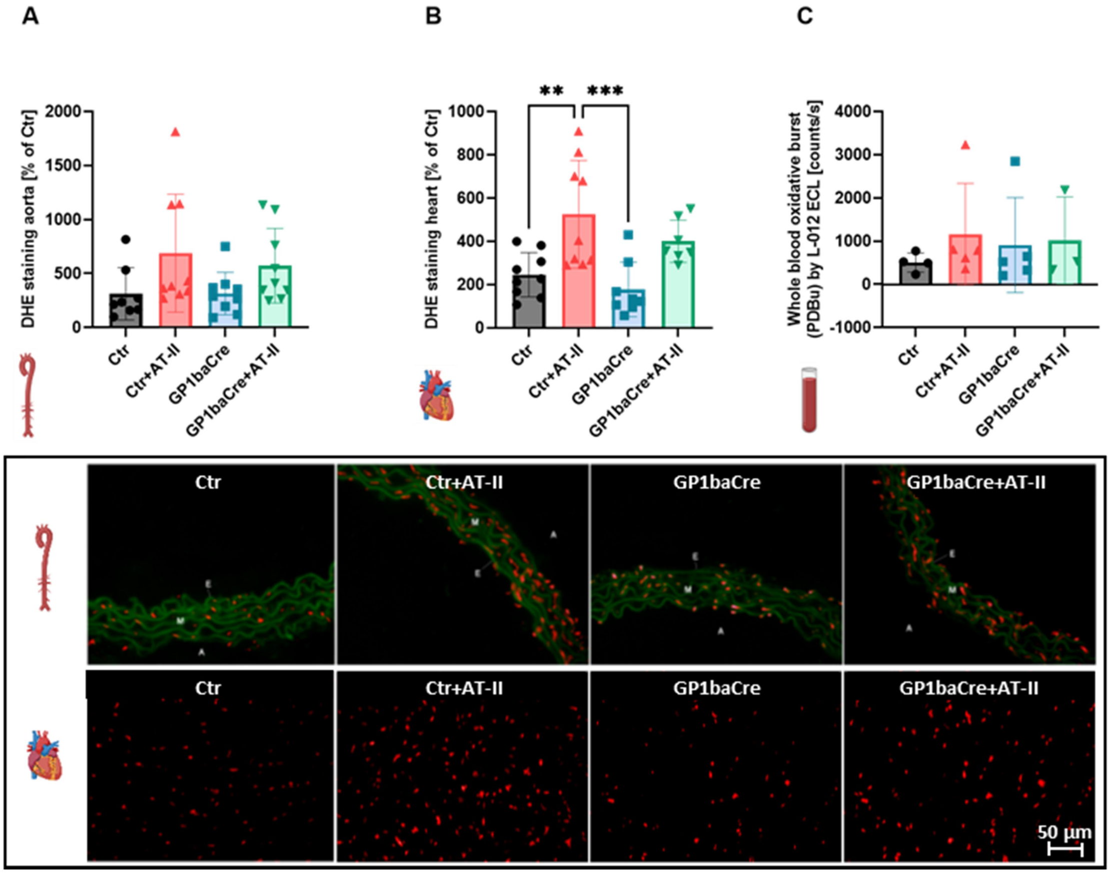

Supplement Figure 4: Platelet-specific CD40L knockout shows no protective effect against oxidative stress in hypertensive mice. Platelet-specific CD40L knockout mice (GP1baCre) and respective control mice (Ctr) were treated with AT-II (1mg/kg/d) for seven days using osmotic minipumps. After treatment, blood was collected, and organs were harvested. Total ROS production was analyzed in cryosections by DHE staining in different tissues: aorta (A) and heart (B). Representative DHE images are shown below the bar graphs. The chemiluminescence of L-012 oxidation was used to analyze the mainly leukocyte-dependent H_2O_2 production in whole blood samples. Blood was previously stimulated with PDBu. Data are presented as mean ± SD of n=9 (A-), n=9 (B), n=3-5 (C) animals. p≤0.05, **p≤0.01, ***p≤0.001 and ****p≤0.0001. One-way ANOVA with Tukey's multiple comparison test (A,B) and two-way ANOVA with Dunnett's multiple comparison test (C) were performed. Icons were created using BioRender.com. Abbreviations: AT-II=angiotensin II, PDBu=phorbol 12,13-dibutyrate.
